# Global geographic patterns of heterospecific pollen receipt help uncover potential ecological and evolutionary impacts across plant communities worldwide

**DOI:** 10.1038/s41598-019-44626-0

**Published:** 2019-05-30

**Authors:** Gerardo Arceo-Gómez, Amelia Schroeder, Cristopher Albor, Tia-Lynn Ashman, Tiffany M. Knight, Joanne M. Bennett, Brian Suarez, Victor Parra-Tabla

**Affiliations:** 1 Department of Biological Sciences, East Tennessee State University, Johnson City, TN USA; 2 Universidad Autónoma de Yucatán, Mérida, Yucatán Mexico; 3 Department of Biological Sciences, University of Pittsburgh, Pittsburgh, PA 15217 USA; 4 Institute of Biology, Martin Luther University Halle-Wittenberg, Am Kirchtor 1, 06108 Halle (Saale), Germany; 5 Department of Community Ecology, Helmholtz Centre for Environmental Research- UFZ, Theodor-Lieser-Straße 4, 06120 Halle (Saale), Germany; 6 German Centre for Integrative Biodiversity Research (iDiv) Halle-Jena-Leipzig, Deutscher Platz 5e, 04103 Leipzig, Germany

**Keywords:** Community ecology, Plant ecology

## Abstract

Species interactions are known to be key in driving patterns of biodiversity across the globe. Plant-plant interactions through heterospecific pollen (HP) transfer by their shared pollinators is common and has consequences for plant reproductive success and floral evolution, and thus has the potential to influence global patterns of biodiversity and plant community assembly. The literature on HP transfer is growing and it is therefore timely to review patterns and causes of among-species variation in HP receipt at a global scale, thus uncovering its potential contribution to global patterns of biodiversity. Here we analyzed published data on 245 species distributed across five continents to evaluate latitudinal and altitudinal patterns of HP receipt. We further analyzed the role of floral symmetry and evolutionary history in mediating patterns of HP receipt. Latitude and elevation affected the likelihood and intensity of HP receipt indicating that HP transfer increases in species-rich communities and in areas with high abundance of vertebrate pollinators. Floral symmetry and evolutionary history determined HP load size across plant communities worldwide. Overall, our results suggest that HP receipt may have the potential to contribute to global geographic patterns of plant diversity by imposing strong selective pressures in species-rich areas across the globe.

## Introduction

Understanding the factors that generate and organize plant diversity in nature has been a long-standing goal in ecology. The importance of indirect plant-plant interactions (i.e. pollinator competition and facilitation) in these two processes has been widely studied, and these have been shown to play a major role^1–5^. In contrast, the ecological and evolutionary consequences of direct plant-plant interactions via heterospecific pollen (hereafter HP) transfer have received considerably less attention. In co-flowering communities high levels of pollinator sharing^6–9^ and heterospecific pollen (hereafter HP) transfer are common (e.g. up to 70% of total pollen load^10–12^). Further evidence shows that HP receipt can decrease plant reproductive success (~20% decrease in seed production) by physically or chemically interfering with conspecific ovule fertilization^10,13^. These negative effects are widespread and have been shown from animal^13^- and wind-dispersed HP donors^14^, even if HP deposition occurs in small amounts (e.g. <5 pollen grains^15^). As a result, HP transfer can be a strong, but perhaps underestimated force driving floral evolution^13,16–18^ and co-flowering community assembly^10,19^. Knowledge on the full extent as well as the causes and consequences of HP receipt is thus a key step towards a more complete understanding of the processes that generate and organize plant diversity in nature.

Increasing evidence suggests that HP transfer is common in natural communities^10,11,20–23^. However, the frequency and intensity of HP receipt varies greatly among plant species (2–100% of flowers, 0.1–74% of total pollen load^10^), and the underlying causes of this variation are largely unknown. To date, this variation has been evaluated among-species within a single plant community or community type^20,21,23,24^. However, patterns and factors mediating HP receipt might also vary across large geographical scales^24^. Knowledge of large-scale geographic patterns of HP transfer dynamics is central for uncovering its potential for contributing to global trends in floral diversification and in mediating patterns of community assembly across plant communities worldwide.

A global pattern of latitudinal and altitudinal variation in plant species diversity has been widely demonstrated, with increasing species diversity with decreasing latitude^25–31^, and at mid to low elevations^26,32–34^. Interestingly, it has also been shown that HP receipt can increase with increasing plant species richness^35^. Thus, it is plausible that plant species growing in communities near the equator and at low elevations, where plant diversity tends to be the highest, will be at greater risk of receiving HP. Evidence of such geographic pattern in HP receipt could suggest a potential role of HP in contributing to global patterns of floral diversification and plant diversity distribution. High levels of HP receipt can select for HP tolerance and avoidance strategies^10,13^, thus imposing strong selective pressures on a wide array of morphological^1,16,17,36–38^ and reproductive traits^18,39,40^. When HP transfer is low, and/or inconsistent, these selective pressures can be predicted to be minimal, while the contrary would be expected when HP transfer is high^10,13^. Higher amounts of HP transfer in regions near the equator could also help explain the global decrease in plant reproductive success observed in these areas^41^. Thus, selection on traits that maximize reproductive success by avoiding or tolerating HP effects can be expected to be stronger in species-rich areas, leading to further diversification and contributing to observed latitudinal patterns of plant biodiversity. Biotic interactions have long been predicted to play a key role in generating latitudinal patterns of biodiversity^42,43^, and plant-plant interactions via HP transfer may not be the exception.

In addition to a plant’s geographical location (latitude and elevation), differences in floral symmetry (radial vs. bilateral), a broad indicator of the level of pollinator generalization, may also contribute to among-species variation in HP receipt^20,21,24^. Plants with radial flowers are expected to be visited by a higher number of pollinator species and to receive larger and more diverse HP loads compared to those with bilateral flowers^21,24^. This prediction has been tested within single communities with inconclusive results^20,21^, and thus whether floral symmetry (pollinator generalization) is a mediator of HP transfer dynamics acting across large geographical scales is not yet tested. It is also possible that other species-shared floral characteristics may influence HP receipt (e.g. stigma size, style exertion^11,23^), and thus closely related species can be expected to receive similar amounts of HP as a result of their shared evolutionary history. However, to our knowledge, the strength of the phylogenetic signal underlying patterns of HP receipt has not been evaluated in any system.

Uncovering the factors that mediate among-species variation in HP receipt at large geographical and evolutionary scales is key if we want to predict its potential ecological and evolutionary consequences, particularly in light of large community-wide changes in pollen transfer dynamics^11^ that result from human disturbances^22,23^. In this study we analyze published data on 245 species to evaluate the effects of latitude, elevation, pollinator generalization and evolutionary history in mediating patterns of HP receipt at a global scale. Specifically we ask the following questions: 1) Does the likelihood and intensity of HP receipt increase with decreasing latitude and/or elevation? 2) Is the likelihood and intensity of HP receipt greater in flowers with radial (generalized) versus bilateral (specialized) symmetry? 3) Does the effect of floral specialization in mediating patterns of HP receipt depend on a plant species’ geographic location (latitude or elevation)? And finally, 4) is there a phylogenetic signal on the likelihood and/or intensity of HP receipt?

## Results

**Figure 1:**
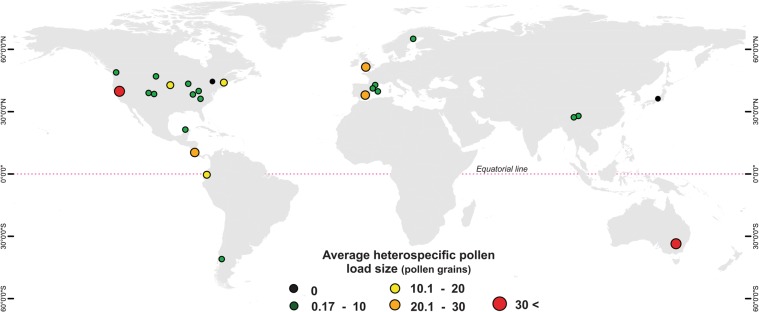
Geographic distribution of the 26 studies from which data on heterospecific pollen receipt was extracted for 245 species. The size and color of each dot represents the average intensity of heterospecific pollen receipt (load size) reported for all species in each study in a given location.

Our dataset included species located in five continents, and their distribution ranged from 63°N to 41°S in latitude and from 0 to 3336 meters above sea level (Fig. 1; Supplementary Data). Average HP load size ranged from 0 to 368.5 pollen grains (mean ± SE; 11.83 ± 2.15).

**Figure 2:**
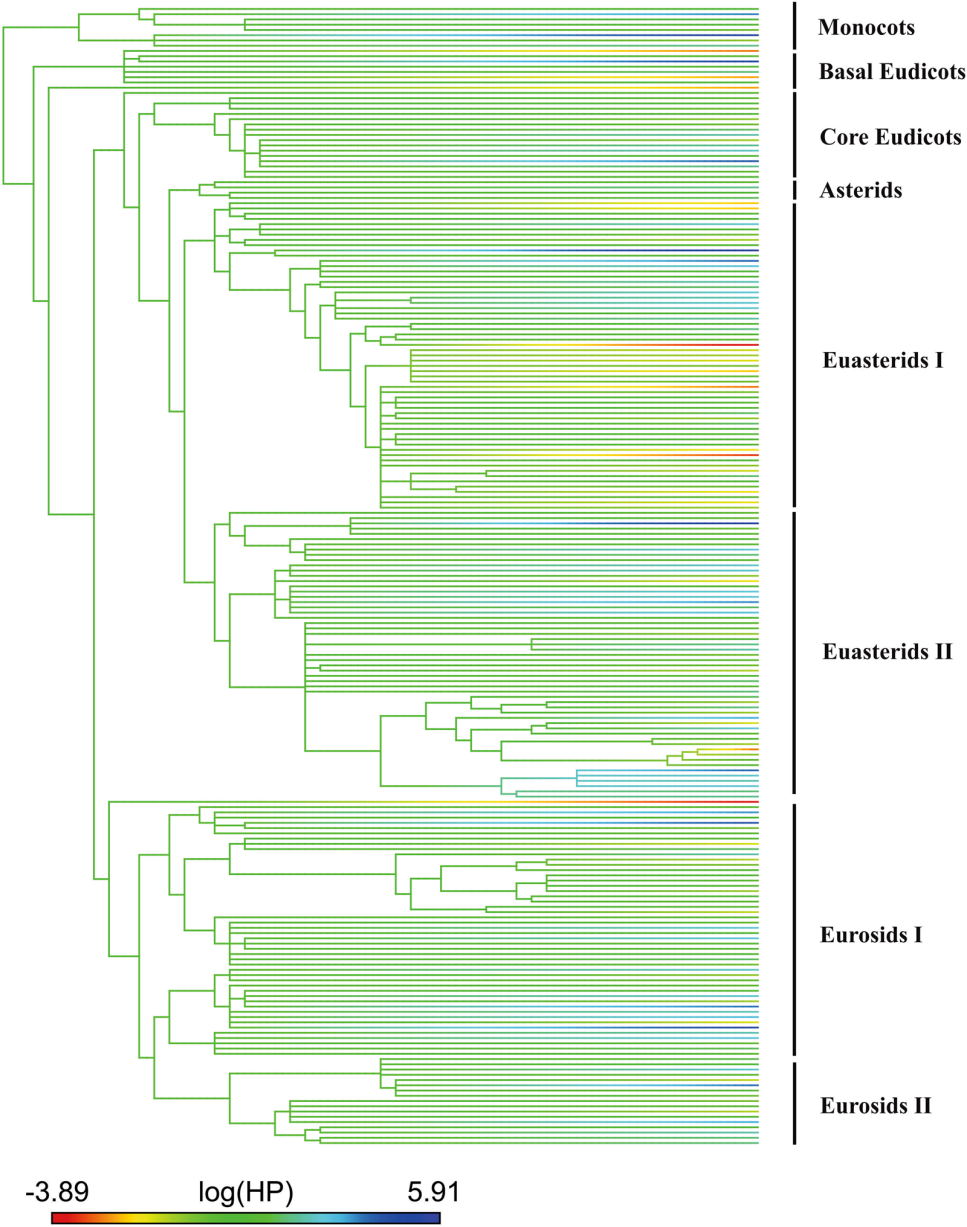
Phylogenetic relationships among the 245 species evaluated in this study. Heterospecific pollen load size (log transformed) for each species is mapped onto the phylogeny and represented by the color of each branch. Phylogenetic relationships were generated from the maximally resolved tree of seed plants within Phylomatic.

**Figure 3:**
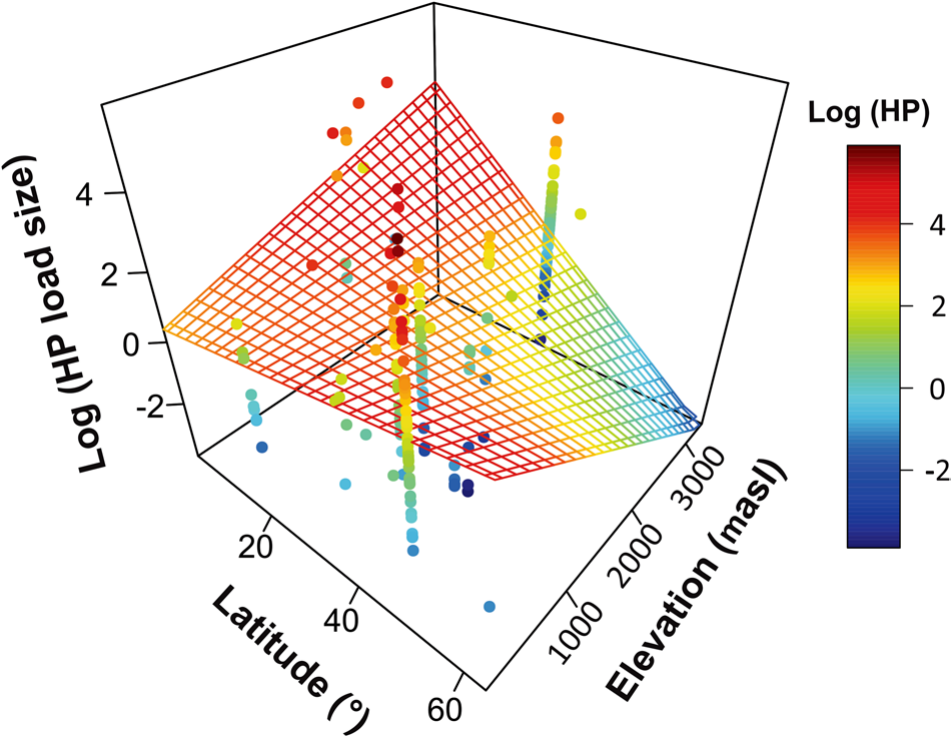
Variation in heterospecific pollen (HP) load size (log transformed) across 217 species according to their altitudinal (meters above sea level) and latitudinal location. Different colors reflect variation in the intensity of HP receipt and the predicted surface indicates geographic areas of high and low intensity of HP receipt.

**Figure 4:**
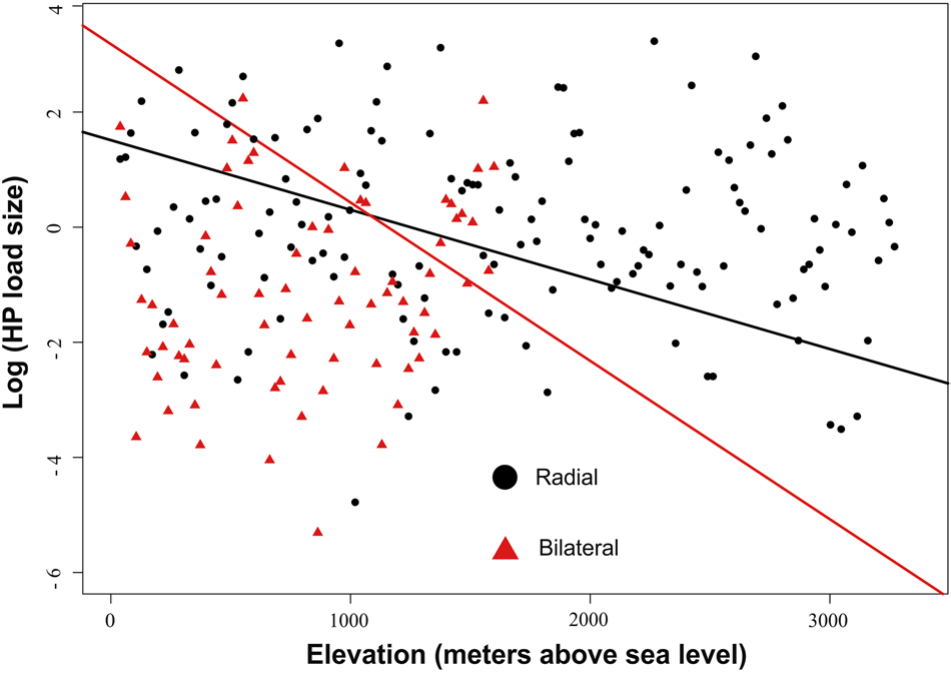
Variation in heterespoecific pollen (HP) load size (log transformed) across 217 species according to their floral symmetry and altitudinal location (meters above sea level). Plant species have been divided based on their floral symmetry into radial (black circles) and bilateral (red triangles) flowers. Both slopes are significant at *P* < 0.05 (see results).

We found a significant phylogenetic signal in HP load size itself (λ = 0.99, K = 0.81, *P* < 0.05 for both; Fig. 2) and in the residuals of the model (λ = 0.7, *P* < 0.01). We also found a significant effect of latitude (*t*_211_ = 2.7, *P* < 0.01) and elevation (*t*_211_ = 3.5, *P* = 0.001) on average HP load size on stigmas. More importantly, however, we found a significant latitude by elevation interaction (*t*_211_ = −3.8, *P* = 0.001; Fig. 3) indicating that both act in combination to influence patterns of HP receipt (in flowers that receive ≥1 HP grain; Fig. 3). Our results also showed that HP load size (≥1 HP grain received) is significantly larger in radial (14.64 ± 3.6) compared to bilateral (11.6 ± 2.8) flowers (*t*_211_ = 3.06, *P < *0.01), however this effect varied with elevation (symmetry by elevation interaction: *t*_211_ = 2.5, *P* = 0.01). While HP receipt increased for both type of flowers (radial and bilateral) with decreasing elevation, the increase was significantly more pronounced for bilateral flowers (Fig. 4). Radial flowers on the other hand, receive more HP than bilateral flowers at high elevations and the increase in HP receipt with decreasing elevation was less steep (Fig. 4). It is important to note that even though the range of elevations was larger for radial compared to bilateral flowers (Fig. 4) this same result was observed when we only considered the altitudinal range for which we have data for both, radial and bilateral flowers (up to 2000 m.a.s.l, *N* = 153; symmetry by elevation interaction, *P* = 0.03). The interaction between latitude and symmetry was not significant (*P* > 0.05) and its exclusion improved the overall fit of the model.

**Figure 5:**
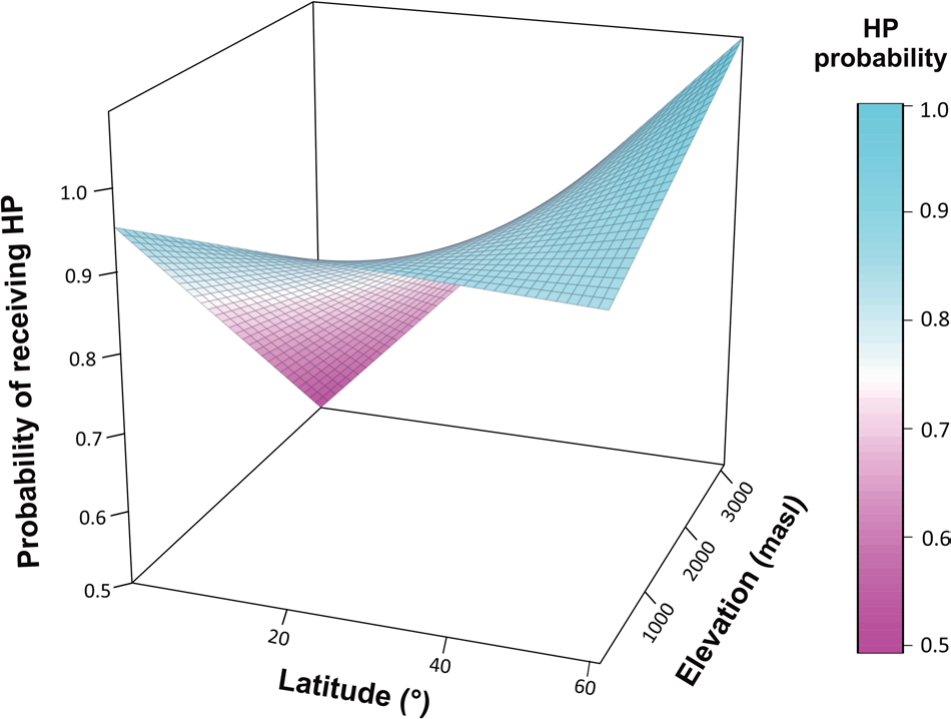
Likelihood of receiving heterospecific pollen (HP) across an altitudinal and latitudinal gradient for the 245 species studied. Different colors reflect variation in the likelihood of HP receipt and the predicted surface indicates geographic areas of high and low likelihood of HP receipt.

There was no phylogenetic signal on the likelihood (presence/absence) of receiving HP itself (D = 0.9, *P* > 0.05) or in the residuals of the model (S2 = 0.25, P > 0.05). Elevation (*Z*_241_ = −2.1, *P* = 0.02) but not latitude (*Z*_241_ = −1.01, *P* = 0.3) significantly affected the likelihood of receiving HP. As before, we found a significant latitude by elevation interaction (z_241_ = 2.06, *P* = 0.03; Fig. 5). Neither floral symmetry nor its interactions with latitude and elevation were significant (*P* > 0.05 for all) and their exclusion improved the overall fit of the model.

## Discussion

Our study revealed a high incidence of HP receipt at a global scale. Of the 245 species evaluated, 88% (217) showed some degree of HP receipt, thus emphasizing the ubiquity of these direct plant-plant interactions in nature^11,22,24^. We also found strong evidence suggesting that species’ geographic location (latitude and elevation) and degree of pollinator generalization (as indicated by flower symmetry) are strong predictors of the likelihood and intensity of HP receipt across plant communities worldwide.

Our results revealed that elevation and latitude interactively influence the intensity of HP receipt. In high latitude sites, HP receipt tends to be higher at low elevations (Fig. 3). This pattern is consistent with the higher diversity of floral resources at these lower elevations^28,30,32,34^, which would lead to higher incidence of pollinator movements, and pollen transfer, between plant species. Indeed, some of our studies that observed high levels of HP transfer^18,35^ come from plant biodiversity hotspots that occur at relatively low elevations and high latitudes, such as the California floristic province^44,45^ and the Mediterranean basin in Europe^46,47^ (Fig. 1). However, at low latitudes (e.g. tropical regions), HP receipt increased with increasing elevation (Fig. 3). Although this pattern seems inconsistent with our initial prediction it may indicate that not only the diversity of the co-flowering community but the composition of the pollinator community plays an important role in mediating patterns of HP receipt^24^. For instance, marked differences in pollinator species composition across altitudinal gradients in the tropics can be expected. Pollinator community composition in tropical rainforests (ca. 1000–5000 m.a.s.l.) can consist of a high diversity and abundance of vertebrate species such as bats, hummingbirds, and even monkeys^17,48–51^. Vertebrate pollinators are typically large in size and are known to carry and deposit large HP loads compared to invertebrate pollinators (e.g. beetles, bees, flies, butterflies) that are more common at low elevations (0–100 m.a.s.l.) in tropical and sub-tropical regions^52–54^. For instance, in a species-rich cloud forest in Ecuador (1300–2300 m.a.s.l.) bat species have been shown to deliver large and diverse HP loads to stigmas^17,55^. Hummingbirds at high elevations (1200 m.a.s.l.) in Costa Rica have also been observed carrying large HP loads of up to six different plant species^51^. Large vertebrate pollinators are less diverse and abundant outside of the tropics^48,56^, and thus the diversity of the co-flowering community may play a larger role in mediating patterns of HP receipt at these higher latitudes. Overall, these results suggest that differences in pollinator body size and foraging behavior may mediate the frequency and amount of HP transfer^24,57^.

Interestingly, even though HP load size increased with elevation in the tropics (Fig. 3), the likelihood of receiving HP was the lowest in this region (Fig. 5). In a similar manner, the likelihood of receiving HP was the highest in high-latitude and low-elevation areas (Fig. 5), where the intensity of HP receipt (HP load size) was the lowest (Fig. 3). These results suggest a potential decoupling of these two processes such that the likelihood of receiving HP and the intensity of HP receipt (HP load size) may be driven by different forces (e.g. random events vs, pollinator size). It is important to point out that in our dataset the number of cases where no HP was received is limited and thus more studies are needed (see below). Nonetheless, our results suggest that distinguishing between these two ecological processes (i.e. likelihood and intensity of HP receipt) is key in order to develop a more predictive understanding of the factors that mediate patterns of HP transfer in nature and how these may vary as a result of human-mediated disturbances^22,23^.

Our findings of higher levels of HP receipt in geographic regions that are predicted to possess high levels of plant diversity, such as in cloud forests and Mediterranean communities, suggest that HP transfer could act as a strong selective force contributing to higher floral diversification in these regions. It has been proposed that HP receipt can lead to the evolution of several HP tolerance and avoidance strategies^10,13^. In fact, HP receipt has been shown to exert a wide variety of selective pressures on plants including morphological traits (e.g. flower size, shape, color, style length and stigma size)^1,10,16,17,36,38^, physiological processes (e. g. pollen tube growth and germination)^10,24^, mating systems^37,39,40^, and flowering phenology^19,58,59^. High levels of HP receipt cannot only impose selection via female fitness but also through male fitness costs^13,17,60^. For instance, it has been shown that HP transfer can be a strong driver of specialization in pollination systems due to high costs of conspecific pollen loss to heterospecific flowers^60^. Thus, it is not unreasonable to expect that higher levels of HP transfer can impose strong and wide-ranging selective pressures that contribute differentially to floral diversification across the globe. Global patterns in HP receipt may also contribute to the high levels of pollen limitation observed in species-rich areas^41^, further strengthening its role in floral evolution and plant community assembly^10,13^ in these regions. HP pollen receipt is thus an untested mechanism that might contribute to overall patterns of pollen limitation. Even though the importance of biotic interactions in contributing to global patterns of diversity has been well documented for a large number of antagonistic and mutualistic interactions^42,43^, the potential for HP transfer interactions in contributing to these patterns has so far been overlooked.

Our results revealed that radial flowers, which are considered more generalized in their pollination system than bilateral flowers^20,21,61^, receive slightly higher amounts of HP, and that HP load size increases with decreasing elevation in both types of flowers (Fig. 4). However, the increase in HP receipt with decreasing elevation was more pronounced for bilateral compared to radial flowers, which tend to receive higher amounts of HP at high elevations (Fig. 4). These results support our prediction of higher HP receipt in generalized, open flowers (radial symmetry), compared to specialized ones (bilateral symmetry). Interestingly, however, our results also suggest that differences in HP receipt between the two flower types (radial vs. bilateral) diminish with decreasing elevation (Fig. 4), where plant diversity tends to be the highest. Overall, these results suggest that floral symmetry (pollinator generalization) may only be a good predictor of HP receipt in plant communities with low species richness such as those at high elevations. These results also suggest that, in low-elevation areas that tend to be species-rich, HP transfer is high across all species regardless of floral symmetry. We also detected a significant phylogenetic signal in the intensity of HP receipt even after accounting for floral symmetry, suggesting that other shared plant traits are still important in mediating the amount of HP received (e.g. stigma area and style exertion^11,23^). On the other hand, we did not detect a phylogenetic signal on the probability of receiving HP. This suggests that whether plants receive HP or not may be strongly influenced by random ‘incidental’ pollination events (e.g. indiscriminate visits to flowers by young bees, misperception of floral cues by inexperienced floral visitors)^24,62^, or by wind-dispersed pollen transfer^63^, thus diminishing the importance of shared floral characteristics.

It is important to acknowledge that even though our findings are consistent with the prediction of higher intensity of HP receipt in areas with high plant diversity and with high abundance of large vertebrate pollinators across the globe, these patterns do not necessarily reflect causation. Experimental assessment of patterns, and the ecological and evolutionary consequences of HP receipt, across gradients of plant^35^ and pollinator diversity would be valuable in confirming the predictions outlined in this study. It is also important to note that even though we observed strong global geographic trends the number of studies documenting patterns of HP receipt is still limited, and strongly biased towards temperate systems (largely concentrated in the United States and Europe; Fig. 1). Studies on HP transfer in diverse regions in Africa and South America are largely underrepresented. Furthermore, in species-rich areas, HP loads may not only be large but also diverse (e.g. >7 species^11^), leading to stronger and synergistic negative effects on plant fitness^64^ with so far unknown consequences. However, we were unable to test for global geographic patterns in the diversity of the HP load given the small number of studies that have reported average or total number of HP donor species per stigma (8 studies). Bias in studies of HP receipt to date is not only geographical but also phylogenetic. For instance, large groups of plants such as monocotyledons have been poorly represented in these studies (Fig. 2). Thus, we stress the need to evaluate patterns of variation in the diversity as well as in the intensity of HP receipt at larger phylogenetic scales, particularly in tropical regions where its ecological and evolutionary consequences might be stronger. Such studies are critical in order to develop a more predictive understanding of the ecological and evolutionary consequences of plant-plant interactions via HP transfer in natural communities across the globe.

## Methods

### Data set

To evaluate patterns of HP receipt at a global scale we collected data from published studies that have reported an average amount of HP on stigmas for one or multiple species in nature. We avoided studies where the diversity and/or composition of the co-flowering community had been experimentally manipulated and only considered studies that reported naturally deposited HP loads. We started by gathering data reported in Appendix S1 in Ashman and Arceo-Gómez^10^. This dataset contained 77 species from 17 studies from 1986 to 2012^10^. We complemented this data by conducting a literature search for studies published between 2012 and 2017 using ISI Web of Science and Google Scholar (key words: heterospecific pollen*, pollen transfer*, pollen load*, pollen*, pollinator sharing*, pollination*). We also included two unpublished datasets, one from the sand dune ecosystem in Yucatan, Mexico (6 species; Parra-Tabla V. unpublished data) and one from a grassland community in Hampton Creek Cove Park Natural Park in Tennessee, USA (26 species; Arceo-Gómez G. unpublished data). In total we compiled information for 279 study cases from 28 different studies distributed across five different continents (Fig. 1). In some cases, data on HP deposition was reported for the same species at the same location multiple times (e.g. different years) and in these cases an average per species at that location was estimated. If the same species was sampled in different geographic locations (i.e. elevation or latitude) one study (species/location combination) was randomly selected for data analyses since phylogenetic models (see below) do not allow for replication of species in the dataset. As a result, 34 observations from 14 species were excluded from this study but the selection of species did not influence the results (Arceo-Gómez G. unpublished data). In total, we analyzed data for 245 species from 26 different studies distributed across five different continents (Fig. 1) and across 52 plant families (Fig. 2; Supplementary Data). For each species, we recorded information on average HP load size (average number of HP grains on stigmas). When data was not available in the text we extracted it from figures using DataThief^65^. When studies only reported the total amount of HP found on stigmas we used sample size data reported to estimate an average. For each species, we also documented its latitudinal (i.e. GPS coordinates) and altitudinal location (meters above sea level). Latitudinal coordinates were converted to decimal degrees and the absolute values were used in analyses. Data on species altitudinal and latitudinal location was gathered from the original study. When information regarding elevation was not provided in the original study, it was estimated using the GPS coordinates reported and topographic data from Google Earth. We also recorded information on floral symmetry and categorized each species as actinomorphic (radial flowers) or zygomorphic (bilateral flowers). Floral symmetry has been commonly used as a broad indicator of pollinator generalization (radial flowers) and specialization (bilateral flowers^20,21,61,66^). When information on floral symmetry for HP recipient species was not available in the original study it was gathered from additional published sources.

### Data analyses

We evaluated the effects of elevation, latitude, floral symmetry and their interaction on the likelihood and intensity (HP load size) of HP receipt using phylogenetic least square models (PGLS) to account for species’ shared evolutionary history^67,68^. For this, we constructed a phylogeny using the most recent megatree in ‘Phylomatic’ (R20160415.new) as our base tree^69^. The final phylogenetic tree was adjusted with branch lengths scaled to time using the BLADJ function in ‘Phylocom’^70^. With this information we estimated phylogenetic signal on the response variables themselves (likelihood and intensity of HP receipt) and on the residuals of each model^68^. For this, we calculated Pagel’s λ^71^ and K-statistic indexes^72,73^ using the function ‘phylosig’ in R^74^. λ is a scaling parameter for the covariance matrix of species traits, relative to the covariance expected under Brownian evolution^73^. K is a scaled ratio of the trait similarity variance among species over the contrasts phylogenetic variance^72,73^. These two indexes vary between zero (no phylogenetic signal) and 1 (complete phylogenetic signal under a Brownian model of trait evolution) and are considered the most robust indexes of phylogenetic signal even in the presence of polytomies^73,75^. We evaluated if phylogenetic signal was significantly different from zero using a likelihood ratio test and null model analysis (1000 randomizations) for ‘λ’ and ‘K’ respectively using Phytools^76^ and the caper packages^77^ in R^74^. If observed phylogenetic signal was not different from zero then a non-phylogenetic model was used in the analysis.

When evaluating effects on the intensity of HP receipt we were interested in evaluating how our predictor variables influenced HP load size and thus we only used the subset of species that receive ≥1 pollen grains for this analysis (*N* = 217). Heterospecific pollen load size was log transformed. The analysis was conducted using the package APE^78^ in R^74^. For evaluating effects on the likelihood of receiving HP we used the entire data set (*N* = 245). For this, we converted data for each species into a binary trait, 0 (no HP received) or 1 (HP received) and used logistic regression^79^ to analyze its relationship with latitude, elevation and floral symmetry. For this particular analysis we used the ‘D-statistic’ and ‘S2’ indexes for estimating phylogenetic signal on the response variable itself and on the residuals of the model, as these are more appropriate for binary data^79,80^. Since no phylogenetic signal was found (see results), we used a non-phylogenetic model to evaluate effects on the likelihood of receiving HP. Estimation of phylogenetic signal was conducted using the package Phylom in R^81^.

We conducted backwards stepwise regression in all the analyses and used Akaike information criterion (AIC) to avoid overparametrization of the models and identify the models with the best fit. We predicted that the likelihood and intensity of HP receipt would decrease at high latitudes and in high elevations and it would be greater for radial compared to bilateral flowers.

## Supplementary information

Dataset 1

## Data Availability

All data generated and analyzed during this study are included in this published article (and its Supplementary Information Files).
